# (*E*)-2-(2*H*-Benzotriazol-2-yl)-4-methyl-6-(phenyl­imino­meth­yl)phenol

**DOI:** 10.1107/S1600536810040468

**Published:** 2010-10-20

**Authors:** Chi-Huan Li, Jing-Kai Su, Chen-Yu Li, Bao-Tsan Ko

**Affiliations:** a Department of Chemistry, Chung Yuan Christian University, Chung-Li 32023, Taiwan

## Abstract

In the title compound, C_20_H_16_N_4_O, the non-H atoms of the benzotriazole ring system and those of the methyl­phenol group are essentially coplanar, with an r.m.s. deviation of 0.004 (2) Å. The mean plane of these atoms forms a dihedral angle of 60.9 (2)° with the phenyl ring. There is an intra­molecular O—H⋯N hydrogen bond between the phenol and benzotriazole groups.

## Related literature

For related structures, see: Chen *et al.* (2010 ▶); Li *et al.* (2009 ▶, 2010 ▶).
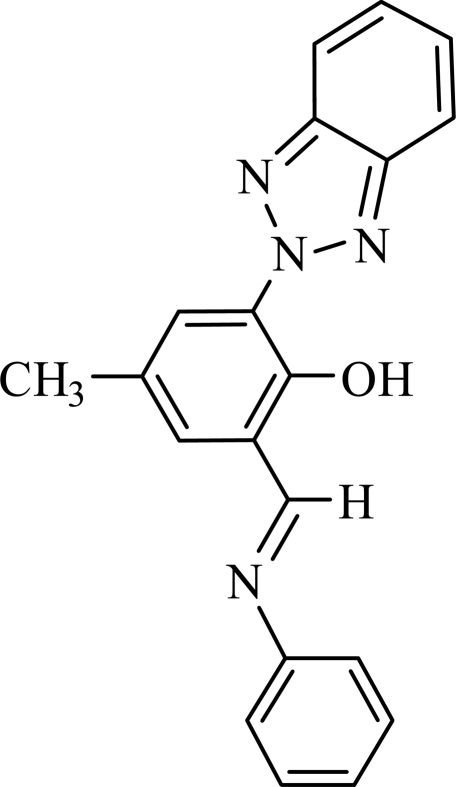


## Experimental

### 

#### Crystal data

C_20_H_16_N_4_O
*M* *_r_* = 328.37
Monoclinic, 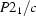

*a* = 15.7279 (5) Å
*b* = 12.3002 (4) Å
*c* = 8.4903 (3) Å
β = 104.842 (1)°
*V* = 1587.70 (9) Å^3^
*Z* = 4
Mo *K*α radiation
μ = 0.09 mm^−1^
*T* = 173 K
0.48 × 0.37 × 0.21 mm

#### Data collection

Bruker APEXII CCD diffractometer
Absorption correction: multi-scan (*SADABS*; Bruker, 2008 ▶) *T*_min_ = 0.959, *T*_max_ = 0.982
15472 measured reflections
3924 independent reflections
2874 reflections with *I* > 2σ(*I*)
*R*_int_ = 0.033

#### Refinement

*R*[*F* ^2^ > 2σ(*F* ^2^)] = 0.054
*wR*(*F* ^2^) = 0.143
*S* = 1.01
3924 reflections
228 parameters
H-atom parameters constrained
Δρ_max_ = 0.41 e Å^−3^
Δρ_min_ = −0.22 e Å^−3^

### 

Data collection: *APEX2* (Bruker, 2008 ▶); cell refinement: *SAINT-Plus* (Bruker, 2008 ▶); data reduction: *SAINT-Plus*; program(s) used to solve structure: *SHELXS97* (Sheldrick, 2008 ▶); program(s) used to refine structure: *SHELXL97* (Sheldrick, 2008 ▶); molecular graphics: *SHELXTL* (Sheldrick, 2008 ▶); software used to prepare material for publication: *SHELXTL*.

## Supplementary Material

Crystal structure: contains datablocks I, global. DOI: 10.1107/S1600536810040468/lh5146sup1.cif

Structure factors: contains datablocks I. DOI: 10.1107/S1600536810040468/lh5146Isup2.hkl

Additional supplementary materials:  crystallographic information; 3D view; checkCIF report

## Figures and Tables

**Table 1:** Hydrogen-bond geometry (Å, °)

*D*—H⋯*A*	*D*—H	H⋯*A*	*D*⋯*A*	*D*—H⋯*A*
O—H0*A*⋯N1	0.84	1.85	2.591 (2)	146

**Fig. 1.**
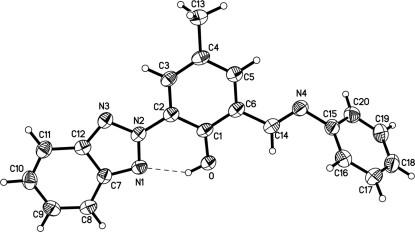
The molecular structure of I with the atom numbering scheme. Displacement ellipsoids are drawn at the 60% probability level.

**Fig. 2.**
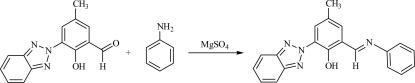
The synthetic procedure of in the preparation of I.

## supplementary crystallographic information

### Comment

Recently, benzotriazole-phenol (BTP-H) derivatives have attracted our attention because the benzotriazole-phenolate group can provide the *N,O*-bidentate chelation to stabilize the transition metal or main group metal complexes. Therefore, our group is interested in the design and synthesis of functionalized benzotriazole-phenolate ligands derived from 4-methyl-2-(2*H*-benzotriazol-2-yl)phenol (*^Me^*BTP-H). For instance, our group has successfully synthesized and structural characterized the methyl ether functionalized BTP derivative *via* etherification derived from *^Me^*BTP-H (Chen *et al.*, 2010). We have also reported the synthesis and crystal structure of a salicylaldehyde group substituted benzotriazole derivative (Li *et al.*, 2010). As part of our goal to prepare *NNO*-tridentate Schiff-base ligands originating from BTP derivatives, we report herein the synthesis and crystal structure of the title compound, (I), which is a potential ligand for the preparation of *NNO*-tridentate Schiff-base zinc (Zn) and magnesium (Mg) complexes.

The molecular structure of (I) shows a 4-methyl-2-((phenylimino)methyl)phenol moiety with a benzotriazole functionalized group on the 6-position (Fig. 1). The non-hydrogen atoms of the benzotriazole ring system and those of the methylphenol group are essentially co-planar with an r.m.s. deviation of 0.004 (2) Å. The mean-plane of these atoms forms a dihedral angle of 60.9 (2)° with the phenyl ring. There is an intramolecular O—H···N hydrogen bond between the phenol and benzotriazole groups (see Table 1). The bond distances of the benzotriazole-phenolate group are similar to those found in the crystal structure of 2-(2*H*-benzotriazol-2-yl)-6-((diethylamino)methyl)-4-methylphenol (Li *et al.*, 2009).

### Experimental

The title compound (I) was synthesized by the following procedure: (Fig. 2): A mixture of aniline (0.27 ml, 3.0 mmol), 3-(2*H*-benzotriazol-2-yl)-2-hydroxy-5-methylbenzaldehyde (0.68 g, 2.7 mmol) and anhydrous MgSO_4_ (2.0 g) were stirred in reflux toluene (20 ml) for 12 h. Volatile materials were removed under vacuum to give yellow solids. Yield: 0.71 g (80%). Yellow crystals were obtained from a saturated Et_2_O solution.

### Refinement

The H atoms were placed in idealized positions and constrained to ride on their parent atoms, with C–H = 0.95 Å with *U_iso_*(H) = 1.2 *U*_eq_(C) for phenyl hydrogen; 0.98 Å with *U*_iso_(H) = 1.5 *U*_eq_(C) for CH_3_ group; 0.95 Å with *U*_iso_(H) = 1.2 *U*_eq_(C) for HC=N group; O–H = 0.84 Å with *U*_iso_(H) = 1.5 *U*_eq_(O).

### Crystal data

**Table:**

C_20_H_16_N_4_O	*F*(000) = 688
*M**_r_* = 328.37	*D*_x_ = 1.374 Mg m^−^^3^
Monoclinic, *P*2_1_/*c*	Mo *K*α radiation, λ = 0.71073 Å
Hall symbol: -P 2ybc	Cell parameters from 5818 reflections
*a* = 15.7279 (5) Å	θ = 2.7–28.2°
*b* = 12.3002 (4) Å	µ = 0.09 mm^−^^1^
*c* = 8.4903 (3) Å	*T* = 173 K
β = 104.842 (1)°	Block, yellow
*V* = 1587.70 (9) Å^3^	0.48 × 0.37 × 0.21 mm
*Z* = 4	

### Data collection

**Table:**

Bruker APEXII CCD diffractometer	3924 independent reflections
Radiation source: fine-focus sealed tube	2874 reflections with *I* > 2σ(*I*)
graphite	*R*_int_ = 0.033
Detector resolution: 8.3333 pixels mm^-1^	θ_max_ = 28.3°, θ_min_ = 2.1°
φ and ω scans	*h* = −20→20
Absorption correction: multi-scan (*SADABS*; Bruker, 2008)	*k* = −16→16
*T*_min_ = 0.959, *T*_max_ = 0.982	*l* = −11→10
15472 measured reflections	

### Refinement

**Table:**

Refinement on *F*^2^	Primary atom site location: structure-invariant direct methods
Least-squares matrix: full	Secondary atom site location: difference Fourier map
*R*[*F*^2^ > 2σ(*F*^2^)] = 0.054	Hydrogen site location: inferred from neighbouring sites
*wR*(*F*^2^) = 0.143	H-atom parameters constrained
*S* = 1.01	*w* = 1/[σ^2^(*F*_o_^2^) + (0.0585*P*)^2^ + 1.1196*P*] where *P* = (*F*_o_^2^ + 2*F*_c_^2^)/3
3924 reflections	(Δ/σ)_max_ < 0.001
228 parameters	Δρ_max_ = 0.41 e Å^−^^3^
0 restraints	Δρ_min_ = −0.22 e Å^−^^3^

### Special details

**Table:**

Experimental. ^1^H NMR (CDCl3, ppm): δ 8.77 (s, 1H, PhN=CH), 7.31-8.03 (m, 11H, PhH), 2.45 (s, 3H, PhCH3).
Geometry. All e.s.d.'s (except the e.s.d. in the dihedral angle between two l.s. planes) are estimated using the full covariance matrix. The cell e.s.d.'s are taken into account individually in the estimation of e.s.d.'s in distances, angles and torsion angles; correlations between e.s.d.'s in cell parameters are only used when they are defined by crystal symmetry. An approximate (isotropic) treatment of cell e.s.d.'s is used for estimating e.s.d.'s involving l.s. planes.
Refinement. Refinement of *F*^2^ against ALL reflections. The weighted *R*-factor *wR* and goodness of fit *S* are based on *F*^2^, conventional *R*-factors *R* are based on *F*, with *F* set to zero for negative *F*^2^. The threshold expression of *F*^2^ > σ(*F*^2^) is used only for calculating *R*-factors(gt) *etc*. and is not relevant to the choice of reflections for refinement. *R*-factors based on *F*^2^ are statistically about twice as large as those based on *F*, and *R*- factors based on ALL data will be even larger.

### Fractional atomic coordinates and isotropic or equivalent isotropic displacement parameters (Å^2^)

**Table:**

	*x*	*y*	*z*	*U*_iso_*/*U*_eq_	
O	0.33970 (8)	0.05791 (10)	0.32102 (16)	0.0282 (3)	
H0A	0.3839	0.0749	0.3960	0.042*	
N1	0.44430 (10)	0.18656 (12)	0.52339 (19)	0.0261 (3)	
N2	0.39775 (10)	0.27027 (12)	0.44505 (18)	0.0237 (3)	
N3	0.42414 (10)	0.36903 (12)	0.50100 (19)	0.0260 (3)	
N4	0.12957 (11)	0.00970 (13)	−0.0541 (2)	0.0300 (4)	
C1	0.29886 (11)	0.14915 (14)	0.2511 (2)	0.0236 (4)	
C2	0.32445 (11)	0.25450 (14)	0.3077 (2)	0.0236 (4)	
C3	0.27847 (12)	0.34491 (14)	0.2315 (2)	0.0255 (4)	
H3B	0.2969	0.4155	0.2713	0.031*	
C4	0.20630 (12)	0.33396 (14)	0.0987 (2)	0.0262 (4)	
C5	0.18144 (12)	0.22937 (15)	0.0425 (2)	0.0263 (4)	
H5A	0.1322	0.2204	−0.0486	0.032*	
C6	0.22639 (12)	0.13762 (14)	0.1156 (2)	0.0250 (4)	
C7	0.50721 (11)	0.23467 (15)	0.6408 (2)	0.0246 (4)	
C8	0.57746 (12)	0.18910 (16)	0.7620 (2)	0.0297 (4)	
H8A	0.5866	0.1128	0.7720	0.036*	
C9	0.63115 (12)	0.26085 (17)	0.8633 (2)	0.0313 (4)	
H9A	0.6789	0.2335	0.9463	0.038*	
C10	0.61819 (12)	0.37458 (16)	0.8491 (2)	0.0317 (4)	
H10A	0.6575	0.4209	0.9230	0.038*	
C11	0.55176 (13)	0.41972 (16)	0.7340 (2)	0.0300 (4)	
H11A	0.5440	0.4963	0.7256	0.036*	
C12	0.49490 (12)	0.34845 (15)	0.6274 (2)	0.0250 (4)	
C13	0.15659 (13)	0.43184 (15)	0.0165 (2)	0.0324 (4)	
H13A	0.1404	0.4780	0.0983	0.049*	
H13B	0.1033	0.4080	−0.0636	0.049*	
H13C	0.1938	0.4732	−0.0385	0.049*	
C14	0.20102 (12)	0.02858 (15)	0.0498 (2)	0.0266 (4)	
H14A	0.2401	−0.0302	0.0872	0.032*	
C15	0.11424 (13)	−0.09878 (15)	−0.1139 (2)	0.0289 (4)	
C16	0.17777 (13)	−0.15712 (16)	−0.1667 (2)	0.0326 (4)	
H16A	0.2324	−0.1241	−0.1664	0.039*	
C17	0.16105 (14)	−0.26315 (17)	−0.2193 (3)	0.0372 (5)	
H17A	0.2043	−0.3025	−0.2561	0.045*	
C18	0.08242 (14)	−0.31249 (17)	−0.2191 (3)	0.0372 (5)	
H18A	0.0722	−0.3862	−0.2520	0.045*	
C19	0.01889 (14)	−0.25433 (17)	−0.1710 (3)	0.0362 (5)	
H19A	−0.0356	−0.2880	−0.1720	0.043*	
C20	0.03324 (13)	−0.14724 (17)	−0.1210 (2)	0.0339 (4)	
H20A	−0.0119	−0.1070	−0.0917	0.041*	

### Atomic displacement parameters (Å^2^)

**Table:**

	*U*^11^	*U*^22^	*U*^33^	*U*^12^	*U*^13^	*U*^23^
O	0.0304 (7)	0.0216 (6)	0.0312 (7)	0.0013 (5)	0.0055 (6)	0.0000 (5)
N1	0.0262 (7)	0.0248 (8)	0.0290 (8)	0.0020 (6)	0.0101 (6)	−0.0001 (6)
N2	0.0269 (7)	0.0220 (7)	0.0255 (8)	−0.0012 (6)	0.0126 (6)	−0.0005 (6)
N3	0.0296 (8)	0.0222 (7)	0.0279 (8)	−0.0019 (6)	0.0104 (6)	−0.0011 (6)
N4	0.0343 (8)	0.0260 (8)	0.0299 (9)	0.0017 (7)	0.0084 (7)	−0.0004 (7)
C1	0.0263 (8)	0.0217 (8)	0.0272 (9)	0.0018 (7)	0.0149 (7)	0.0012 (7)
C2	0.0241 (8)	0.0243 (9)	0.0256 (9)	0.0001 (7)	0.0123 (7)	0.0005 (7)
C3	0.0297 (9)	0.0207 (8)	0.0300 (10)	−0.0001 (7)	0.0149 (8)	−0.0006 (7)
C4	0.0299 (9)	0.0235 (9)	0.0296 (10)	0.0045 (7)	0.0158 (8)	0.0031 (7)
C5	0.0267 (8)	0.0276 (9)	0.0266 (9)	0.0006 (7)	0.0105 (7)	0.0003 (7)
C6	0.0282 (9)	0.0230 (9)	0.0286 (9)	−0.0001 (7)	0.0157 (8)	−0.0002 (7)
C7	0.0257 (8)	0.0254 (9)	0.0266 (9)	−0.0022 (7)	0.0138 (7)	−0.0032 (7)
C8	0.0328 (9)	0.0253 (9)	0.0338 (10)	0.0040 (8)	0.0136 (8)	0.0013 (8)
C9	0.0261 (9)	0.0394 (11)	0.0289 (10)	0.0031 (8)	0.0080 (8)	0.0023 (8)
C10	0.0308 (9)	0.0351 (10)	0.0316 (10)	−0.0087 (8)	0.0123 (8)	−0.0055 (8)
C11	0.0344 (10)	0.0253 (9)	0.0336 (10)	−0.0037 (8)	0.0144 (8)	−0.0017 (8)
C12	0.0267 (8)	0.0260 (9)	0.0261 (9)	0.0008 (7)	0.0138 (7)	0.0026 (7)
C13	0.0345 (10)	0.0262 (9)	0.0356 (11)	0.0042 (8)	0.0070 (8)	0.0029 (8)
C14	0.0301 (9)	0.0231 (9)	0.0288 (10)	0.0014 (7)	0.0115 (8)	0.0003 (7)
C15	0.0354 (10)	0.0258 (9)	0.0231 (9)	0.0009 (8)	0.0032 (8)	0.0013 (7)
C16	0.0301 (9)	0.0352 (10)	0.0317 (10)	−0.0023 (8)	0.0064 (8)	−0.0003 (9)
C17	0.0395 (11)	0.0364 (11)	0.0363 (11)	0.0050 (9)	0.0108 (9)	−0.0071 (9)
C18	0.0468 (12)	0.0299 (10)	0.0323 (11)	−0.0038 (9)	0.0055 (9)	−0.0045 (9)
C19	0.0354 (10)	0.0382 (11)	0.0335 (11)	−0.0092 (9)	0.0059 (9)	−0.0025 (9)
C20	0.0303 (10)	0.0380 (11)	0.0332 (10)	0.0018 (8)	0.0076 (8)	−0.0027 (9)

### Geometric parameters (Å, °)

**Table:**

O—C1	1.352 (2)	C8—H8A	0.9500
O—H0A	0.8400	C9—C10	1.414 (3)
N1—N2	1.338 (2)	C9—H9A	0.9500
N1—C7	1.348 (2)	C10—C11	1.354 (3)
N2—N3	1.331 (2)	C10—H10A	0.9500
N2—C2	1.427 (2)	C11—C12	1.404 (3)
N3—C12	1.358 (2)	C11—H11A	0.9500
N4—C14	1.260 (2)	C13—H13A	0.9800
N4—C15	1.426 (2)	C13—H13B	0.9800
C1—C6	1.404 (3)	C13—H13C	0.9800
C1—C2	1.405 (2)	C14—H14A	0.9500
C2—C3	1.392 (2)	C15—C20	1.394 (3)
C3—C4	1.386 (3)	C15—C16	1.394 (3)
C3—H3B	0.9500	C16—C17	1.382 (3)
C4—C5	1.393 (2)	C16—H16A	0.9500
C4—C13	1.506 (2)	C17—C18	1.378 (3)
C5—C6	1.391 (2)	C17—H17A	0.9500
C5—H5A	0.9500	C18—C19	1.373 (3)
C6—C14	1.468 (2)	C18—H18A	0.9500
C7—C12	1.413 (3)	C19—C20	1.385 (3)
C7—C8	1.418 (3)	C19—H19A	0.9500
C8—C9	1.364 (3)	C20—H20A	0.9500
			
C1—O—H0A	109.5	C11—C10—H10A	118.8
N2—N1—C7	103.56 (15)	C9—C10—H10A	118.8
N3—N2—N1	116.36 (14)	C10—C11—C12	117.10 (18)
N3—N2—C2	121.88 (14)	C10—C11—H11A	121.4
N1—N2—C2	121.76 (14)	C12—C11—H11A	121.4
N2—N3—C12	103.27 (14)	N3—C12—C11	130.60 (17)
C14—N4—C15	117.45 (16)	N3—C12—C7	108.41 (16)
O—C1—C6	118.06 (16)	C11—C12—C7	120.98 (18)
O—C1—C2	123.58 (16)	C4—C13—H13A	109.5
C6—C1—C2	118.35 (16)	C4—C13—H13B	109.5
C3—C2—C1	120.56 (17)	H13A—C13—H13B	109.5
C3—C2—N2	119.07 (16)	C4—C13—H13C	109.5
C1—C2—N2	120.37 (15)	H13A—C13—H13C	109.5
C4—C3—C2	121.34 (17)	H13B—C13—H13C	109.5
C4—C3—H3B	119.3	N4—C14—C6	122.78 (17)
C2—C3—H3B	119.3	N4—C14—H14A	118.6
C3—C4—C5	117.88 (16)	C6—C14—H14A	118.6
C3—C4—C13	121.25 (17)	C20—C15—C16	119.25 (18)
C5—C4—C13	120.86 (17)	C20—C15—N4	118.99 (17)
C6—C5—C4	122.06 (17)	C16—C15—N4	121.76 (17)
C6—C5—H5A	119.0	C17—C16—C15	119.81 (19)
C4—C5—H5A	119.0	C17—C16—H16A	120.1
C5—C6—C1	119.80 (16)	C15—C16—H16A	120.1
C5—C6—C14	120.93 (17)	C18—C17—C16	120.77 (19)
C1—C6—C14	119.24 (16)	C18—C17—H17A	119.6
N1—C7—C12	108.40 (16)	C16—C17—H17A	119.6
N1—C7—C8	130.60 (17)	C19—C18—C17	119.52 (19)
C12—C7—C8	121.00 (17)	C19—C18—H18A	120.2
C9—C8—C7	116.32 (18)	C17—C18—H18A	120.2
C9—C8—H8A	121.8	C18—C19—C20	120.85 (19)
C7—C8—H8A	121.8	C18—C19—H19A	119.6
C8—C9—C10	122.26 (19)	C20—C19—H19A	119.6
C8—C9—H9A	118.9	C19—C20—C15	119.70 (19)
C10—C9—H9A	118.9	C19—C20—H20A	120.1
C11—C10—C9	122.34 (19)	C15—C20—H20A	120.1
			
C7—N1—N2—N3	−0.34 (18)	N1—C7—C8—C9	179.37 (18)
C7—N1—N2—C2	178.92 (14)	C12—C7—C8—C9	0.3 (2)
N1—N2—N3—C12	0.31 (18)	C7—C8—C9—C10	−0.2 (3)
C2—N2—N3—C12	−178.95 (14)	C8—C9—C10—C11	−0.1 (3)
O—C1—C2—C3	−178.92 (15)	C9—C10—C11—C12	0.2 (3)
C6—C1—C2—C3	0.4 (2)	N2—N3—C12—C11	179.15 (18)
O—C1—C2—N2	0.7 (2)	N2—N3—C12—C7	−0.15 (18)
C6—C1—C2—N2	−179.96 (15)	C10—C11—C12—N3	−179.33 (17)
N3—N2—C2—C3	−0.9 (2)	C10—C11—C12—C7	−0.1 (3)
N1—N2—C2—C3	179.87 (15)	N1—C7—C12—N3	−0.04 (19)
N3—N2—C2—C1	179.46 (15)	C8—C7—C12—N3	179.22 (15)
N1—N2—C2—C1	0.2 (2)	N1—C7—C12—C11	−179.42 (15)
C1—C2—C3—C4	0.3 (3)	C8—C7—C12—C11	−0.2 (3)
N2—C2—C3—C4	−179.37 (15)	C15—N4—C14—C6	177.26 (16)
C2—C3—C4—C5	−0.6 (3)	C5—C6—C14—N4	−13.4 (3)
C2—C3—C4—C13	179.92 (16)	C1—C6—C14—N4	168.64 (17)
C3—C4—C5—C6	0.2 (3)	C14—N4—C15—C20	132.8 (2)
C13—C4—C5—C6	179.72 (16)	C14—N4—C15—C16	−47.7 (3)
C4—C5—C6—C1	0.5 (3)	C20—C15—C16—C17	−2.4 (3)
C4—C5—C6—C14	−177.48 (16)	N4—C15—C16—C17	178.13 (18)
O—C1—C6—C5	178.60 (15)	C15—C16—C17—C18	−0.6 (3)
C2—C1—C6—C5	−0.8 (2)	C16—C17—C18—C19	2.2 (3)
O—C1—C6—C14	−3.4 (2)	C17—C18—C19—C20	−0.7 (3)
C2—C1—C6—C14	177.22 (15)	C18—C19—C20—C15	−2.2 (3)
N2—N1—C7—C12	0.21 (17)	C16—C15—C20—C19	3.8 (3)
N2—N1—C7—C8	−178.95 (17)	N4—C15—C20—C19	−176.73 (18)

### Hydrogen-bond geometry (Å, °)

**Table:**

*D*—H···*A*	*D*—H	H···*A*	*D*···*A*	*D*—H···*A*
O—H0A···N1	0.84	1.85	2.591 (2)	146
